# 1841. Younger age and female gender are associated with delayed antibiotics in pediatric sepsis

**DOI:** 10.1093/ofid/ofad500.1669

**Published:** 2023-11-27

**Authors:** Sarah B Kandil, Seohyuk Lee, Richard Feinn, Thomas S Murray

**Affiliations:** Yale School of Medicine, New Haven, Connecticut; Yale University School of Medicine, New Haven, Connecticut; Quinnipiac, Hamden, Connecticut; Yale University School of Medicine, New Haven, Connecticut

## Abstract

**Background:**

Sepsis is a leading cause of pediatric mortality, with nearly 5,000 children dying annually.(Weiss, Fitzgerald et al. 2015) Clinical advances in care bundles to improve sepsis recognition and timely treatment have demonstrated improved outcomes.(Lane, Funai et al. 2016) Recently, literature suggests an association between pediatric sepsis mortality and various social determinants of health, including race and socioeconomic status.(Reddy, Badolato et al. 2022, Li, Ng et al. 2023) It is unclear what may contribute to these disparities; therefore, we chose to evaluate if race, ethnicity or other factors resulted in delayed antibiotic administration.

**Methods:**

This retrospective cohort review of a single center quality improvement pediatric sepsis dataset included admitted patients with a blood culture and intravenous antibiotics within 4 hours and a hospital diagnosis of sepsis. Patients admitted to the neonatal intensive care unit were excluded. The primary outcome was antibiotic administration > 60 minutes. Exposures of interest included age, gender, race, ethnicity, language, sepsis severity level, ICU admission and mortality. Analysis included descriptive statistics and Chi-Square testing.

**Results:**

From 2016 – 2023 there were 322 patients [43% White, 19% Black, 38% Other (Asian, multiple racial, or unavailable)], with 61% identified as non-Hispanic and 39% Hispanic. English was the primary language (83%) and there were 52% females, and 48% males. Overall, 58% of patients required intensive level care and 6.8% died. The mean time to antibiotics was 75 minutes. There was no statistical significance when evaluating if race, ethnicity, language, or sepsis severity contributed to antibiotic delays of > 60 minutes. Factor associated with a statistically significant delay in antibiotic administration were: mortality (68.2 died % vs 31.8% survived; p < 0.003) and female gender (46% female vs 29% male ; p < 0.002); regardless of sepsis severity and infants < 1 year (59% < 1 year, 32%, 1-17 years, and 31 % >18 years; p< 0.001).

**Conclusion:**

Inequities exist in the timely delivery of antibiotics for younger infants and female patients with an admitting diagnosis of sepsis. Further investigation is warranted to identify drivers for these inequities.

**Disclosures:**

**All Authors**: No reported disclosures

